# New living evidence resource of human and non-human studies for early intervention and research prioritisation in anxiety, depression and psychosis

**DOI:** 10.1136/bmjment-2023-300759

**Published:** 2023-06-08

**Authors:** Andrea Cipriani, Soraya Seedat, Lea Milligan, Georgia Salanti, Malcolm Macleod, Janna Hastings, James Thomas, Susan Michie, Toshi A Furukawa, David Gilbert, Karla Soares-Weiser, Carmen Moreno, Stefan Leucht, Matthias Egger, Parisa Mansoori, James M Barker, Spyridon Siafis, Edoardo Giuseppe Ostinelli, Robert McCutcheon, Simonne Wright, Matilda Simpson, Olufisayo Elugbadebo, Virginia Chiocchia, Thomy Tonia, Rania Elgarf, Ayse Kurtulmus, Emily Sena, Ouma Simple, Niall Boyce, Sophie Chung, Anjuli Sharma, Miranda Wolpert, Jennifer Potts, Julian H Elliott

**Affiliations:** 1 Department of Psychiatry, University of Oxford, Oxford, UK; 2 Oxford Precision Psychiatry Lab, NIHR Oxford Health Biomedical Research Centre, Oxford, UK; 3 Oxford Health NHS Foundation Trust, Warneford Hospital, Oxford, UK; 4 South African Medical Research Council/Stellenbosch University Extramural Genomics of Brain Disorders Unit, Department of Psychiatry, Stellenbosch University, Cape Town, South Africa; 5 MQ, Mental Health Research, London, UK; 6 Institute of Social and Preventive Medicine, University of Bern, Bern, Switzerland; 7 Centre for Clinical Brain Sciences, University of Edinburgh, Edinburgh, UK; 8 Institute for Implementation Science in Health Care, Faculty of Medicine, University of Zurich, Zurich, Switzerland; 9 School of Medicine, University of St. Gallen, St. Gallen, Switzerland; 10 EPPI Centre, Social Research Institute, University College London, London, UK; 11 UCL Centre for Behaviour Change, University College London, London, UK; 12 Department of Health Promotion and Human Behavior, Kyoto University Graduate School of Medicine / School of Public Health, Kyoto, Japan; 13 Chair, GALENOS Global Experiential Advisory Board, InHealth Associates, London, UK; 14 Cochrane, London, UK; 15 Department of Child and Adolescent Psychiatry, Institute of Psychiatry and Mental Health, Hospital General Universitario Gregorio Marañón, IiSGM, CIBERSAM, ISCIII, School of Medicine, Universidad Complutense, Madrid, Spain; 16 Department of Psychiatry and Psychotherapy, School of Medicine, Technical University of Munich, Munich, Germany; 17 Centre for Infectious Disease Epidemiology and Research, Faculty of Health Sciences, University of Cape Town, Cape Town, South Africa; 18 Population Health Sciences, Bristol Medical School, University of Bristol, Bristol, UK; 19 F1000, Research Ltd, London, UK; 20 Department of Psychosis Studies, Institute of Psychiatry, Psychology and Neuroscience, King’s College, London, UK; 21 Department of Psychiatry, University of Ibadan, Ibadan, Nigeria; 22 Department of Psychiatry, Istanbul Medeniyet University, Turkey, Turkey; 23 College of Health Sciences, Makerere University, Kampala, Uganda; 24 Wellcome, London, UK; 25 Cochrane Australia, School of Public Health and Preventive Medicine, Monash University, Melbourne, Victoria, Australia; 26 Future Evidence Foundation, Melbourne, Victoria, Australia

**Keywords:** Anxiety disorders, Depression & mood disorders, Schizophrenia & psychotic disorders, Child & adolescent psychiatry, Adult psychiatry

## Abstract

In anxiety, depression and psychosis, there has been frustratingly slow progress in developing novel therapies that make a substantial difference in practice, as well as in predicting which treatments will work for whom and in what contexts. To intervene early in the process and deliver optimal care to patients, we need to understand the underlying mechanisms of mental health conditions, develop safe and effective interventions that target these mechanisms, and improve our capabilities in timely diagnosis and reliable prediction of symptom trajectories. Better synthesis of existing evidence is one way to reduce waste and improve efficiency in research towards these ends. Living systematic reviews produce rigorous, up-to-date and informative evidence summaries that are particularly important where research is emerging rapidly, current evidence is uncertain and new findings might change policy or practice. Global Alliance for Living Evidence on aNxiety, depressiOn and pSychosis (GALENOS) aims to tackle the challenges of mental health science research by cataloguing and evaluating the full spectrum of relevant scientific research including both human and preclinical studies. GALENOS will also allow the mental health community—including patients, carers, clinicians, researchers and funders—to better identify the research questions that most urgently need to be answered. By creating open-access datasets and outputs in a state-of-the-art online resource, GALENOS will help identify promising signals early in the research process. This will accelerate translation from discovery science into effective new interventions for anxiety, depression and psychosis, ready to be translated in clinical practice across the world.

## Problem

Anxiety, depression and psychosis are among the top leading causes of the global burden of disease.^1^ Current prevention approaches and treatments have an important role but even in combination are not effective for everyone.^2^ For decades now, there have been no major breakthroughs in diagnosing or treating these conditions. We urgently need better interventions and care to prevent illness, alleviate symptoms, decrease distress and promote recovery.

To deliver impactful advances, mental health science must address the following barriers to progress:

1. The field is fragmented, with polarised arguments too often dependent on ideology, not evidence.^3^ *We need to provide a foundation of common ground based on the best available data from experiments and observations to drive discovery of new and repurposed treatments*.
2. Mental health interventions are frequently targeted at reducing the burden of symptoms rather than addressing the causes of mental health conditions and/or their persistence.^4^ *We need to understand these underlying mechanisms better and develop targeted interventions*.
3. Current research is not always focused on the outcomes that matter most to affected individuals and communities.^5^ *We need to coproduce recommendations and priorities for future research through an equal partnership with people with lived experience of mental health conditions*.
4. The high volume of scientific publication makes it increasingly difficult to appraise and translate research advances into clinical practice in a timely and rigorous way.^6^ *We must provide evidence-synthesis strategies that allow researchers and the wider community to harness prior work fully*.

## Our vision

To tackle these challenges, we have launched a research programme called Global Alliance for Living Evidence on aNxiety, depressiOn and pSychosis (GALENOS, https://galenos.org.uk/). GALENOS aims to coproduce a continuously updated and trustworthy synthesis of the scientific literature focused on the mechanisms underlying anxiety, depression and psychosis, and their prevention and treatment. This will allow the mental health community to better identify future research questions that could materially advance the field and deliver impact for patients and services. We aim to create a freely accessible, state-of-the-art resource to accelerate discovery science, identify effective new interventions, develop better tools and foster personalised care.

We will publish *a series of living systematic reviews*.^7^ These reviews will focus on the most promising scientific findings (from basic laboratory and animal research to clinical studies in humans), assessing early pharmacological treatments, social and psychological interventions, and diagnostic, prognostic, and predictive tools in depression, anxiety and psychosis across the life span.

**Figure 1:**
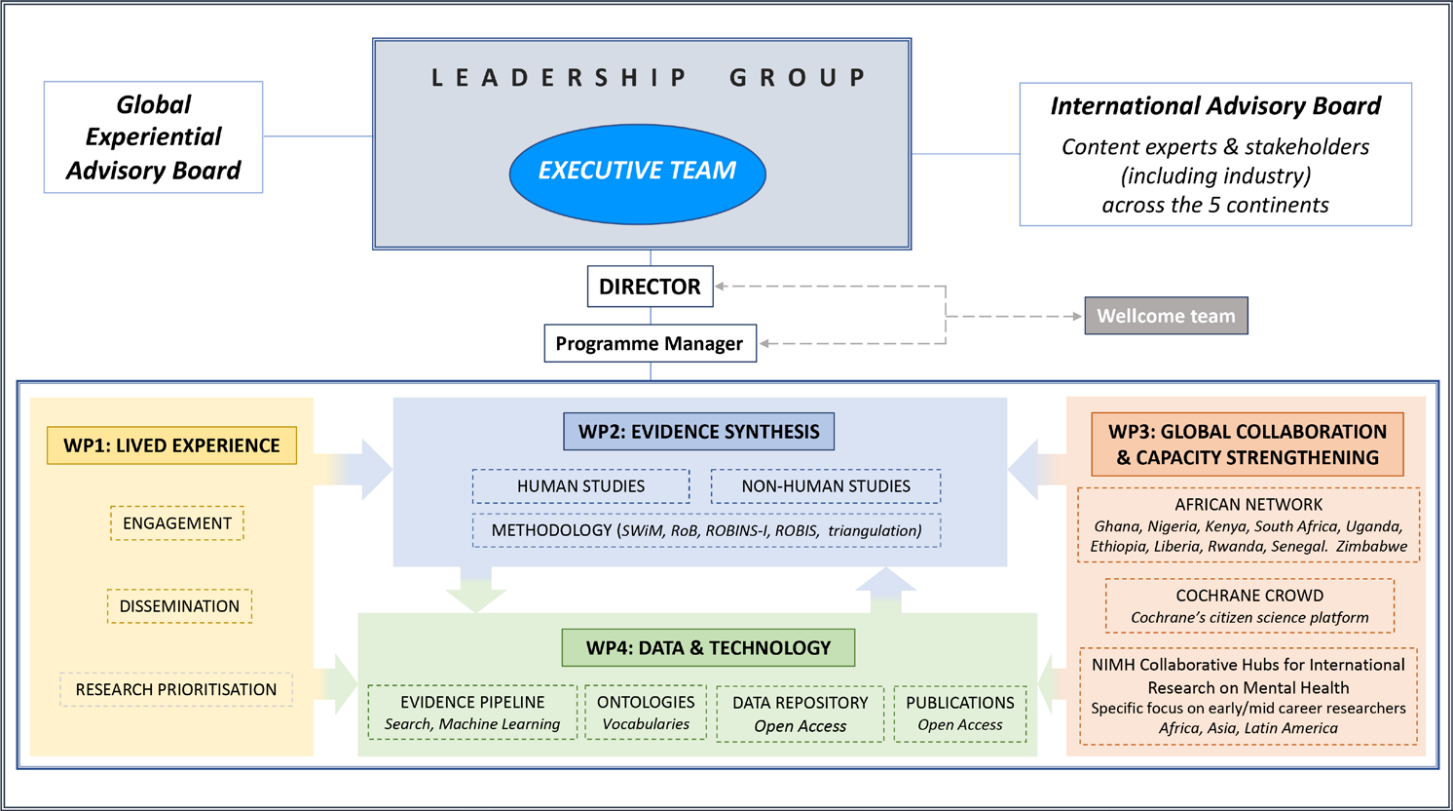
Global Alliance for Living Evidence on Anxiety, Depression and Psychosis organisational structure: leadership group, advisory boards and four interconnected WPs. WP, work package.

GALENOS will be conducted by a global, multidisciplinary consortium, including mental health clinicians, researchers, evidence synthesis methodologists, statisticians, and experts in technology and data science. It will include wider engagement with and insight from people with mental health conditions and is built on four pillars (figure 1).

### Coproduction with experts through experience (WP1: lived experience)

Equal partnerships with people with lived experience of mental health conditions at all levels are central to GALENOS, from choosing the topics to be addressed to key roles in governance and strategic leadership. People with experience of mental health conditions will be *experiential advisors* in GALENOS, strategic partners who bring a unique ‘experiential lens’ and toolkit of common and reflected-upon qualities from their experiences.^8^ At the governance level, there will be a global experiential advisory board, an international and demographically diverse group that provides oversight of the engagement strategy.

### Summarising various sources of evidence (WP2: evidence synthesis)

We will analyse evidence from a variety of study designs to answer our research questions including preclinical experiments,^9^ observational studies and clinical trials, and identify knowledge gaps. We will apply suitable statistical models for meta-analysis to synthesise the data, if appropriate.^10 11^ We will develop and use a framework to evaluate the biases and credibility of the conclusions of each systematic review.^12–14^ Human and non-human studies will produce different types of evidence, with systematic errors and biases that are largely unrelated. By triangulating their results based on the direction and strength of the evidence, we aim to gain additional insights and more comprehensive conclusions.^15^

### Curation by a global community (WP3: global collaboration and capacity strengthening)

**Figure 2:**
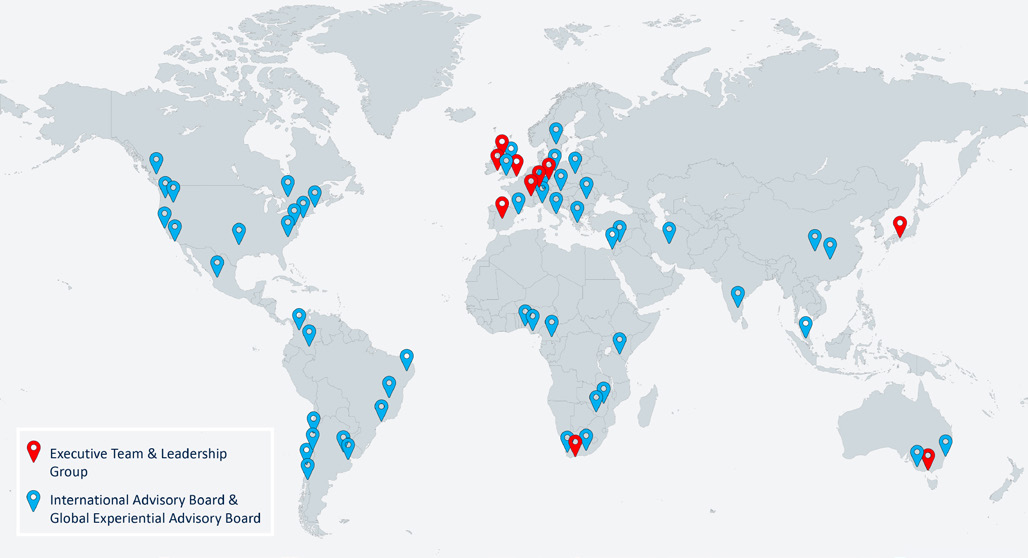
Global Alliance for Living Evidence on Anxiety, Depression and Psychosis: a global resource curated by a global community.

Our vision is a global resource *curated by* and *relevant to* a global community. The GALENOS team brings diverse perspectives from different geographical regions and an extensive network of collaborators from 35 countries (figure 2). It includes researchers across various aspects of mental health science (from biological to social sciences), affected communities and practitioners. Our responsibility is to generate a positive, equal and thriving environment for knowledge translation through rapid and effective use of our extensive partnerships and networks straddling high-income, middle-income and low-income contexts. We aim to build authentic, long-term engagement across all aspects of the project, creating optimal conditions for effective dissemination. We will train a global crowd of researchers who will join our collaborative volunteer effort to identify the most relevant evidence. We will also consult with an international advisory board of experts and stakeholders from academia, healthcare institutions and industry to be sure we cover the most important aspects of mental health research and collect constructive feedback in a collaborative effort.

### Maximising the value of data (WP4: data and technology)

We will produce an open-access and interactive platform. All the protocols and living systematic reviews (with user-friendly summaries) will be published as Diamond Open Access in Wellcome Open Research, in collaboration with F1000 (https://www.f1000.com/). Outputs from this project, including methodological papers, will be made available through an interactive online gateway, and we will also publish in traditional journals, as appropriate. Updated datasets with extracted study characteristics, study results, risk of bias assessments and results of meta-analyses will be made available according to findability, accessibility, interoperability and reusability (FAIR) principles.^16^ Drawing on the information from GALENOS, we will engage with globally diverse research and affected communities to create *maps of proposed research priorities* to inform the field and support future funder decision making.

To help organise the GALENOS programme of research, we will build a comprehensive classification of meaningful categories of the researched subject matter and their relationships—an *ontology*—for the mental health domain.^17^ Using the methodology developed in the Human Behaviour-Change Project,^18^ which draws extensively from stakeholder feedback, we will extend its Behaviour Change Intervention Ontology^19^ to the full scope of mental health research. Where possible, the terms used to classify our research will be linked with other controlled vocabularies, for example, Medical Subject Headings. The approach will enable us to organise and link knowledge across academic disciplines and topic domains and share GALENOS data and other resources in standardised, FAIR forms that facilitate the discovery of personalised and effective new interventions. The mental health ontology and algorithms will learn from the growing evidence repository. We can thus accumulate knowledge about which aspects of the evidence can be generalised from larger evidence bases, primarily in the Global North, to underserved communities, mainly in the Global South. This will help us bridge the gap in mental health services between different world regions. Furthermore, we will employ technologies drawing from the machine-readable ontology to help speed up the identification of relevant research and maximise the efficiency of managing the large and growing evidence base. Human inputs will focus on tasks that cannot yet be carried out automatically with sufficient accuracy.

GALENOS is supported financially by Wellcome. Wellcome’s mental health programme has a vision of a world in which no one is held back by mental health problems, with a mission to drive a step change in early intervention for anxiety, depression and psychosis. Wellcome aims to achieve this by gaining a better understanding of how the brain, body and environment interact in the development and resolution of these conditions, so that we can spot potential points for early intervention; discovering more effective means of identifying and grouping people with—or at risk of—these conditions so that we can provide more timely and personalised interventions; and finding new and improved pharmacological and non-pharmacological ways of intervening. The diverse and often siloed mental health research communities need to both collaborate and coalesce with a shared focus and understanding of the state of the evidence on key areas of emerging potential. We therefore anticipate that GALENOS’s output will be of high value in both Wellcome’s mission and the work of the wider mental health research community.

## Conclusions

Fresh thinking and global action are required to improve the return on society’s investments in mental health research and reduce the burden of mental health conditions. GALENOS will create and maintain a free and open-access global resource for everyone concerned with mental health, seeking new insights that accelerate progress in developing new tools, treatments and systems for the prevention and early intervention of anxiety, depression and psychosis. To foster innovation, we need to make the best use of existing data and build systems that enable dynamic, reliable and efficient representation, and thereby understanding of mental health research findings. We will be traversing uncharted territory and call on those who are interested in joining us in this important endeavour. Everyone is invited to apply if they would like to become part of our crowd, register on the website for updates, follow GALENOS on social media and use the products of this project. It is a unique opportunity to improve the global research enterprise that must deliver better outcomes for people with anxiety, depression and psychosis. We look forward to demonstrating the usefulness of this approach, and we hope that it will be adopted by other funders and colleagues, and for other human health issues.
